# Evaluating the clinical utility of *Aspergillus*, *Mucorales,* and *Nocardia* bronchoalveolar PCRs for the diagnosis of invasive pulmonary infections in patients with hematological malignancies

**DOI:** 10.1128/jcm.01355-24

**Published:** 2025-01-16

**Authors:** Varshini Gali, Rakan Al-Ghanamah, Katie Finnigan, Or Kalchiem-Dekel, Mini Kamboj, Tobias M. Hohl, N. Esther Babady, Genovefa A. Papanicolaou, Yeon Joo Lee

**Affiliations:** 1 Department of Medicine, Weill Cornell Medical College 5803 , New York, New York, USA; 2 Infectious Diseases Service, Department of Medicine, Memorial Sloan Kettering Cancer Center 571678 , New York, New York, USA; 3 Pulmonary Service, Department of Medicine, Memorial Sloan Kettering Cancer Center 5803 , New York, New York, USA; 4 Clinical Microbiology Service, Department of Pathology and Laboratory Medicine, Memorial Sloan Kettering Cancer Center 5803 , New York, New York, USA; University of Calgary, Calgary, Alberta, Canada

**Keywords:** invasive pulmonary infections, Aspergillus PCR, Mucorales PCR, Nocardia PCR, bronchioalveolar lavage, invasive pulmonary aspergillosis, pulmonary nocardiosis, pulmonary mucormycosis, hematopoietic stem cell transplant, hematologic malignancies

## Abstract

Invasive pulmonary infections are a significant cause of morbidity and mortality in patients with hematological malignancies and hematopoietic stem cell transplantation (HCT) recipients. A delay in identifying a causative agent may result in late initiation of appropriate treatment and adverse clinical outcomes. We examine the diagnostic utility of PCR-based assays in evaluating invasive pulmonary infections from bronchoalveolar lavage (BAL). Patients with hematological malignancies and HCT recipients who underwent bronchoscopy with BAL from January 2020 to January 2024 for unexplained pulmonary infiltrates and had ≥1 PCR targeting *Aspergillus*, *Mucorales*, or *Nocardia* (Eurofins-Viracor, KS) were reviewed. Testing for microbiology and pathology except BAL PCRs to identify the etiology of pulmonary infiltrate was defined as standard-of-care. Invasive fungal diseases were defined as per European Organization for Research and Treatment of Cancer and the Mycoses Study Group Education and Research Consortium (EORTC/MSGERC) 2020 guidelines. Pulmonary nocardiosis was defined by a combination of clinical, radiographic, and microbiologic criteria. Of 134 patients, 77 were HCT recipients, and 70% were on antifungal agents. Thirty-two were diagnosed with infection with one of the three target pathogens, including 20 with probable or proven invasive pulmonary aspergillosis (IPA), seven with mucormycosis, and three with nocardiosis. For IPA, 19 were diagnosed by standard-of-care, and one (5%) was solely diagnosed by *Aspergillus* PCR. *Mucorales* PCR was positive in three of seven cases of proven mucormycosis, but the cultures were negative in all. All three nocardiosis cases were detected by PCR and culture. In our cohort, PCR targeting *Mucorales* and *Nocardia* can improve the early detection of invasive pulmonary infection, whereas *Aspergillus* PCR has a low added value when done in conjunction with standard-of-care, including BAL galactomannan.

**IMPORTANCE:**

Invasive pulmonary infections are a significant cause of morbidity and mortality in immunocompromised patients. Timely diagnosis of invasive pulmonary infection reduces the time to targeted treatment initiation and improves clinical outcomes. The recent European Organization for Research and Treatment of Cancer and the Mycoses Study Group Education and Research Consortium (EORTC/MSGERC) update included the addition of serum or bronchoalveolar lavage (BAL) PCR as a method to determine probable *Aspergillus* disease. This reflects an increased utilization of PCR-based assays in the diagnosis of fungal diseases. Although PCR assays for *Aspergillus* diagnosis have been well characterized in the literature, their additive clinical utility in conjunction with BAL galactomannan index measurements remains unclear. Moreover, only a few reports characterize the analytic and clinical performance of *Mucorales* and *Nocardia* PCR.

## INTRODUCTION

Invasive pulmonary infections are a significant cause of morbidity and mortality in immunocompromised patients (1–3). Patients with hematologic malignancies and hematopoietic stem cell transplant (HCT) recipients are at high risk for opportunistic fungal and atypical bacterial infections, such as *Aspergillus, Mucorales*, and *Nocardia*. Invasive *Aspergillus* has a reported incidence of up to 2%–12% in patients with hematologic malignancies and recipients of HCT (3–6). *Mucorales*, such as *Rhizopus*, *Mucor*, and *Licthemia*, are less common but are associated with high mortality rates of 37%–80% (2, 7, 8). Mortality rates for nocardiosis in cancer patients or HCT recipients have been reported to be as high as 60%–63% (9, 10).

Timely diagnosis of invasive pulmonary infection reduces time to efficacious treatment initiation and improves clinical outcomes (11, 12). In a single-center study, delayed amphotericin B-based therapy for mucormycosis resulted in a 2-fold increase in mortality (11). The goal of definitive early diagnosis is often not achievable due to the limitations of conventional diagnostic methods, including tissue biopsy and histology, microbial culture, and serologic biomarkers (13). Biomarker diagnosis is currently lacking for mucormycosis. Conventional diagnostic modalities have low sensitivity, whereas fungal biomarkers lack specificity. 1, 3-β-d-glucan (BDG) can be falsely elevated in patients with immunoglobulin therapy (14, 15). There is a cross-reactivity of *Fusarium species* with galactomannan (16). Thus, implementing rapid and reliable methods of diagnosing invasive pulmonary infection remains an area of profound interest.

The recent European Organization for Research and Treatment of Cancer and the Mycoses Study Group Education and Research Consortium (EORTC/MSGERC) update included the addition of serum or bronchoalveolar lavage (BAL) PCR as a method to determine probable *Aspergillus* disease. This reflects an increased utilization of PCR-based assays in the diagnosis of fungal diseases (17). For *Mucorales* infection, the gold standard for diagnosis remains direct visualization or growth of the fungus in tissue biopsy or BAL. The gold standard for *Nocardia* infection diagnosis is culture; however, recovery may require up to 3 weeks of culture (18, 19). Although PCR assays for *Aspergillus* diagnosis have been well characterized in the literature with reported sensitivity and specificity of BAL *Aspergillus* PCR up to 57%–93% and 92%–99%, respectively (13), their additive clinical utility in conjunction with BAL galactomannan index measurements remains unclear. Moreover, only a few reports characterize the analytic and clinical performance of *Mucorales* and *Nocardia* PCR (20).

In this study, we evaluated the utility of commercially available *Aspergillus*, *Mucorales*, and *Nocardia* PCR assays in the rapid diagnosis of invasive pulmonary infections from BAL samples in patients with hematologic malignancies and recipients of HCT.

## MATERIALS AND METHODS

### Study cohort

We conducted a retrospective cohort study at Memorial Sloan Kettering Cancer Center (MSK, a 564-bed tertiary care cancer center that performs over 600 HCTs annually). The study cohort included adult and pediatric patients with hematologic malignancy or HCT recipients who underwent bronchoscopy with BAL between January 2020 and January 2024. Patients were included if BAL PCR testing was sent for *Aspergillus*, *Mucorales*, and/or *Nocardia* to Eurofins-Viracor (Lexena, KS) for suspected invasive pulmonary infection based on clinical and radiographic presentation.

### Definitions

“Proven” and “Probable” invasive fungal diseases for *Aspergillus* and *Mucorales* were defined as per the EORTC/MSGERC guidelines (17). Pulmonary nocardiosis was defined by correlating clinical findings, culture from BAL, pathology if a biopsy was performed, and radiology images. Patients who underwent multiple bronchoscopy sample acquisitions were counted once if the bronchoscopy procedures were performed <6 weeks apart. If BAL PCRs or BAL galactomannan (GMA) were discrepant between the procedures, bronchoscopy with positive results was included. Testing for microbiology and pathology other than BAL PCRs to identify the etiology of pulmonary infiltrate was defined as standard-of-care testing.

### Diagnostic tests

#### Bronchoscopy specimens

Standard-of-care testing for bronchoscopy-acquired specimens in patients with hematologic malignancies and HCT recipients includes bacterial, fungal, and mycobacterial cultures, *Pneumocystis jirovecii* (PJP) PCR, *Aspergillus* galactomannan antigen enzyme immunoassay (EIA) (Bio-Rad Platella *Aspergillus* Ag with a positive index cutoff of >0.5), respiratory pathogen PCR panel (BioFire Respiratory 2.1. Panel), and cytology. Additional testing included fine needle aspirate specimens and histopathologic evaluation of tissue biopsy specimens, if available. PCR for *Aspergillus*, *Mucorales*, and/or *Nocardia* were sent for testing at Eurofins-Viracor reference laboratories (https://www.eurofins-viracor.com). The BAL *Aspergillus* qualitative PCR panel is comprised of three real-time PCR assays: a Pan-*Aspergillus* assay that detects all *Aspergillus* species, an *Aspergillus fumigatus* assay, and an *Aspergillus terreus* assay. The BAL *Mucorales* qualitative PCR targets 18 species from the order *Mucorales* including: *Absidia coerulea*, *Lichtheimia corymbifera*, *Absidia glauca*, *Apophysomyces elegans*, *Cunninghamella echinulata*, *Cunninghamella elegans*, *Mucor circinelloides*, *Mucor flavus*, *Mucor hiemalis*, *Mucor indicus*, *Mucor mucedo*, *Mucor racemosus*, *Mucor ramosissimus*, *Rhizomucor pusillus*, *Rhizopus microsporus*, *Rhizopus oryzae*, *Rhizopus stolonifer*, and *Saksenaea vasiformis*. The BAL *Nocardia* qualitative PCR targets 11 *Nocardia* species (*Nocardia cyriacigeorgica*, *Nocardia asteroides complex*, *Nocardia nova complex*, *Nocardia transvalensis*, *Nocardia wallacei*, *Nocardia brasiliensis*, *Nocardia pseudobrasiliensis*, *Nocardia africana*, *Nocardia abscessus*, *Nocardia otitidiscaviarum*, and *Nocardia farcinica*). The turnaround time is within 12–24 h from receipt of specimen.

### Serum galactomannan

Serum *Aspergillus* galactomannan antigen EIA (Bio-Rad Platella *Aspergillus* Ag with a positive index cutoff of >0.5) was tested at the clinician’s discretion. Serum galactomannan was captured if tested within 2 weeks of the index bronchoscopy procedure.

#### Data collection

Medical and pharmacy records were obtained from the MSK electronic medical records system.

### Statistical methods

Categorial variables are presented as counts and percentages, whereas continuous variables are presented as medians and interquartile range (IQR). Statistical tests were performed using IBM SPSS (Version 29).

## RESULTS

### Study patients

Out of the 142 study participants, eight underwent multiple bronchoscopy procedures within the prespecified timeframe of 6 weeks. For these eight participants, one representative bronchoscopy was included in the final analysis. The final analysis included a total of 134 patients (Table 1). The median age was 62.5 years (interquartile range [IQR]: 45.8, 73), 36% were female, and 77 were HCT recipients (82% allogeneic). At the time of bronchoscopy, 13 (9.7%) were on liposomal amphotericin ± mold active azole ± echinocandin, 73 (54.5%) were on mold active azoles ± echinocandin, and 8 (5.9%) were on echinocandin. Forty patients (29.9%) were not on antifungal agents within 2 weeks of bronchoscopy. The absolute neutrophil count was <0.5 K/µL for ≥2 weeks preceding the bronchoscopy in 47 patients (35.1%). All patients underwent bronchoscopy with BAL, and 39% underwent concurrent transbronchial lung biopsy.

**TABLE 1:** Baseline characteristics^*c*^

Characteristic	Value *N* = 134 (%)
Age, median (range) (IQR), years	62.5 (9, 85) (45.8, 73)
Sex, male	86 (64.2%)
Underlying diseases	
History of allogeneic HCT^*a*^	63 (47.0%)
History of autologous HCT	9 (6.8%)
Acute leukemia	38 (28.4%)
Myelodysplastic syndrome	2 (1.5%)
Lymphoma	11 (8.2%)
Chronic lymphocytic leukemia	7 (5.2%)
Polycythemia vera	1 (0.7%)
Multiple myeloma	3 (2.2%)
Absolute neutrophil count <0.5 K/µL at the time of bronchoscopy	48 (35.8%)
Median absolute neutrophil counts (IQR)	2 (0.1, 4.13)
Absolute neutrophil count <0.5 K/µL ≥2 weeks prior to bronchoscopy	47 (35.1%)
On prednisone equivalent ≥20 mg ≥2 weeks prior to bronchoscopy	15 (11.2%)
On antifungal agent within 14 days prior to bronchoscopy	94 (70.1%)
Liposomal amphotericin ± mold active azoles^*b*^ ± echinocandin	13
Mold active azoles ± echinocandin	73
Echinocandin	8

^*a*^ One patient with sickle cell disease received allogeneic HCT.

^*b*^ Mold active azoles include isavuconazole, posaconazole, and voriconazole.

^*c*^ N, number; HCT, hematopoietic cell transplant; IQR, interquartile range.

### *Aspergillus* PCR from BAL

**Fig 1:**
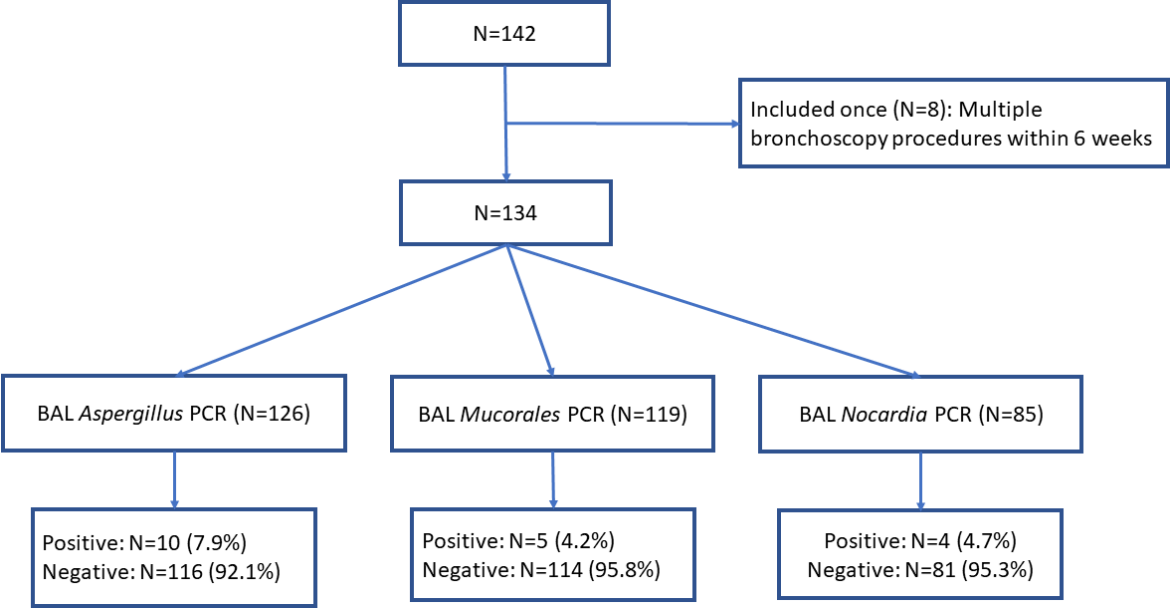
Study population.

Of 134 patients, BAL fluid was tested for *Aspergillus* PCR in 126 (94%) patients and for galactomannan in 129 (96%) patients (Fig. 1). Serum galactomannan was tested in 125 patients; however, in six patients, the test was not performed within 2 weeks of bronchoscopy. Of 119 patients with serum galactomannan tested within 2 weeks of bronchoscopy, the assay was positive in 10 (8.4%).

**Fig 2:**
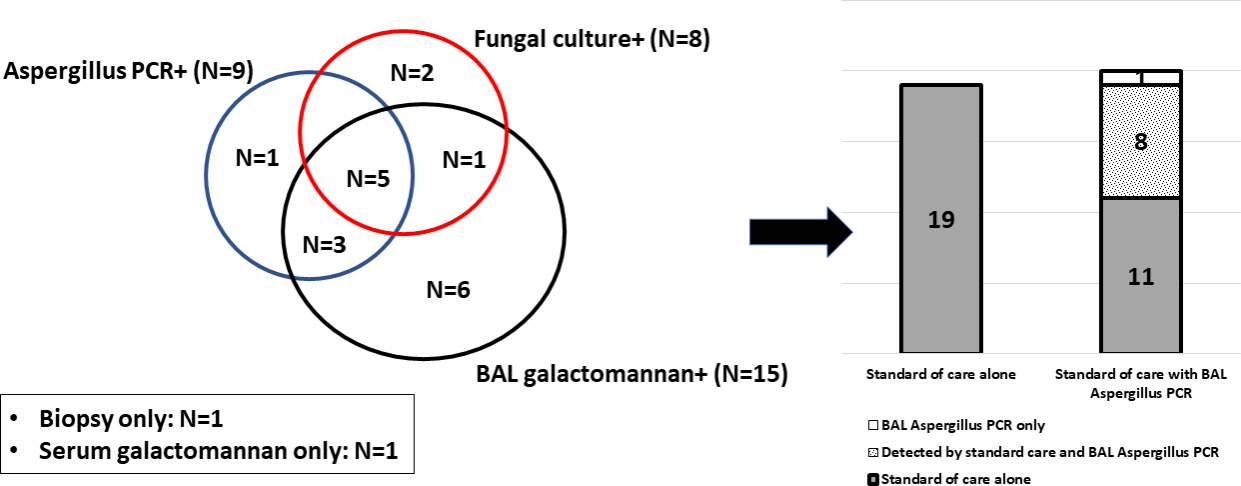
Patients with proven or probable invasive pulmonary *Aspergillus* by diagnosis methods.

Overall, 20 participants met the criteria for invasive pulmonary aspergillosis (IPA); three for “proven” and 17 for “probable” IPA (Table 2). Ten patients tested positive for BAL *Aspergillus* PCR, 9 (90%) of whom were diagnosed with probable or proven IPA. The remaining patient with a positive *Aspergillus* PCR was positive for BAL galactomannan with an index value of 0.94; however, the clinical picture was not compatible with IPA, and therefore, this patient did not receive antifungal treatment. On follow-up 2 months after bronchoscopy, the pulmonary nodules remained stable off antifungal treatment. Of 20 patients with probable or proven IPA, 19 were diagnosed by the standard of care diagnostic methods, of which BAL *Aspergillus* PCR was positive in eight. One patient (case #3), with proven IPA diagnosed by pathology, had negative BAL galactomannan, negative *Aspergillus* PCR, negative serum galactomannan, and negative culture. In one patient with probable IPA (case #11), only the serum galactomannan was positive. One patient was diagnosed with probable IPA by BAL *Aspergillus* PCR (case #10). The additive diagnostic value of BAL *Aspergillus* PCR for probable and proven IPA was 1 of 20 (5%) (Fig. 2).

**TABLE 2:** Clinical characteristics of patients with probable and proven invasive pulmonary aspergillosis^*c*^

Case	Age/sex	Underlying disease	EORTC/MSGERC classification	Biopsy	BAL GMA (value)	Serum GMA (value)	BAL *Aspergillus* PCR	BAL fungal culture	Dignosis method of IPA
1	M/29	ALL	Proven	Fungal infection invaded necrotic tissue, c/w *Aspergillus*	Positive (>6.84)	Positive (3.53)	Positive^*b*^	*A. fumigatus*	Biopsy, serum and BAL GMA, PCR, culture
2	M/67	Lymphoma, s/p alloHCT	Proven	Fungal elements embedded with necrotic tissue, c/w *Aspergillus*	Positive (9.33)	Negative	Positive^*b*^	*A. fumigatus*	Biopsy, BAL GMA, PCR, culture
3	M/48	AML, s/p alloHCT	Proven	Fungal hyphae in granulation tissue and focal necrosis, c/w *Aspergillus*	Negative	Negative	Negative	Negative	Biopsy
4	F/67	AML	Probable	N/A	Positive (3.5)	Negative	Positive^*b*^	*A. niger, Scedosporium boydii*	BAL GMA, PCR, culture
5	M/70	AML, s/p alloHCT	Probable	N/A	Positive (1.79)	Positive (2.43)	Negative	*A. ustus, Scedosporium boydii*	BAL and serum GMA, culture
6	M/71	CLL, s/p alloHCT	Probable	N/A	Positive (>4.39)	Negative	Negative	Negative	BAL GMA
7	F/60	ALL, s/p alloHCT	Probable	Angioinvasive fungal infection c/w mucormycosis^*a*^	Positive (>4.89)	Positive (3.99)	Positive^*b*^	Negative	BAL and serum GMA, PCR
8	F/24	AML, s/p alloHCT	Probable	N/A	Positive (>7.87)	Positive (5.11)	Positive^*b*^	Negative	BAL and serum GMA, PCR
9	F/64	CLL	Probable	N/A	Positive (7.03)	Negative	Positive	Negative	BAL GMA and PCR
10	N/77	AML	Probable	N/A	Negative	Negative	Positive	*Purpureocillium lilacinum*	PCR
11	F/70	AML	Probable	N/A	Negative	Positive (2.31)	Negative	Negative	Serum GMA
12	N/34	ALL	Probable	N/A	Positive (0.67)	Positive (1.34)	Negative	Negative	Serum GMA
13	M/62	Myelodysplastic syndrome	Probable	N/A	Negative	Negative	Negative	*A. fumigatus*	Culture
14	M/75	ALL	Probable	N/A	Positive (4.26)	Positive (1.57)	Positive^*b*^	*A. fumigatus*	BAL and serum GMA, PCR, culture
15	F/21	AML, s/p alloHCT	Probable	N/A	Positive (1.95)	Positive (>6.99)	Positive^*b*^	*A. flavus*	BAL and serum GMA, PCR, culture
16	F/76	Lymphoma	Probable	Non-diagnostic fragment	Positive (1.98)	Positive (0.56)	Negative	Negative	BAL GMA
17	M/62	Lymphoma	Probable	Organizing pneumonia	Negative	Negative	Negative	*A. fumigatus*	Culture
18	F/72	Myeloma, s/p autoHCT	Probable	N/A	Positive (2.16)	Negative	Negative	Negative	BAL GMA
19	M/27	ALL, s/p alloHCT	Probable	N/A	Positive (1.17)	Negative	Negative	Negative	BAL GMA
20	M/58	CLL, s/p alloHCT	Probable	N/A	Positive (1.93)	Negative	Negative	Negative	BAL GMA

^*a*^ Patients had proven pulmonary mucormycosis in addition to probable invasive pulmonary aspergillosis.

^*b*^ Cases with BAL *Aspergillus fumigatus* PCR positive.

^*c*^ M, male; F, female,; ALL, acute lymphoblastic leukemia; s/p, status post; alloHCT, allogeneic HCT; AML, acute myelogenous leukemia; CLL, chronic lymphocytic leukemia; autoHCT, autologous HCT; N/A, not applicable; BAL, bronchoalveolar lavage; GMA, galactomannan; IPA, invasive pulmonary aspergillosis.

Among nine patients with BAL *Aspergillus* PCR positive, BAL *A. fumigatus* PCR was positive in six patients; fungal culture positive with *A. fumigatus* positive in three patients, *Aspergillus niger* in one patient, and two were no growth. None were positive for BAL *A. terreus* PCR.

Five of nine patients (55.6%) with positive BAL *Aspergillus* PCR and six of 15 (40%) with positive BAL galactomannan had concurrent growth in culture (Fig. 2). Among the PCR positive-culture positive-BAL galactomannan-positive samples, the following species were isolated: *A. fumigatus* (*n* = 3), *A. niger* (*n* = 1), and *A. flavus/oryzae* (*n* = 1). In one patient with BAL galactomannan positive-culture-positive sample, *A. ustus* grew. *A. fumigatus* grew in two patients with positive culture only.

### *Mucorales* PCR from BAL

BAL *Mucorales* PCR was tested in 119 (88.8%) study participants (Fig. 1). Seven of the 119 (5.9%) patients met the criteria for proven invasive mucormycosis. None grew in culture, and three of seven (43%) with proven pulmonary mucormycosis were *Mucorales* PCR positive. Fungal element was present in case #2, but the culture was negative (Table 3). BAL *Mucorales* PCR was positive in two patients with possible invasive fungal disease (cases #8 and #9), but the biopsy was not performed due to refractory thrombocytopenia. Table 3 shows a clinical description of the seven proven invasive mucormycosis and two with positive BAL *Mucorales* without biopsy.

**TABLE 3:** Clinical characteristics from patients with proven pulmonary mucormycosis and positive BAL *Mucorales* PCR

Case	Age/sex	Underlying disease	EORTC/MSGERC classification	Radiologic signs	Biopsy	Tissue fungal culture	BAL fungal culture	BAL *Mucorales* PCR	Additional tests to diagnose mucormycosis
1	M/85	Lymphoma	Proven	Consolative mass with central low attenuation	Fungal infection within necrotic tissue c/w mucormycosis	Negative	Negative	Negative	
2	M/68	Polycythemia vera	Proven	Consolidation with internal cavitation	Angioinvasive fungal infection c/w mucormycosis	Fungal element present, culture-negative	Negative	Positive	
3	M/68	AML, s/p alloHCT	Proven	Consolidation with ground glass opacities	Angioinvasive fungal infection c/w mucormycosis	Negative	Negative	Positive	
4	F/60	ALL, s/p alloHCT	Proven	Multifocal consolidations	Angioinvasive fungal infection c/w mucormycosis	Negative	Negative	Negative	
5	M/24	AML, s/p alloHCT	Proven	Consolidation with ground glass opacities	Fungal infection c/w mucormycosis	Negative	Negative	Negative	
6	M/55	AML, s/p alloHCT	Proven	Groundglass consolidation with reverse halo appearance	Angioinvasive fungal infection c/w mucormycosis	Negative	Negative	Positive	*Rhizopus oryzae* by microbial cell-free DNA^*a*^
7	M/68	AML, s/p alloHCT	Proven	Consolidation with central lucency	Fungal infection with tissue invasion and angioinvasion c/w mucormycosis	Negative	Negative	Negative	*Rhizopus oryzae* by panfungal PCR^*b*^
8	M/67	AML, s/p alloHCT	Possible	Mass like consolidaiton with ground glass opacities	Not performed due to thrombocytopenia	Not done	Negative	Positive	
9	M/66	AML, s/p alloHCT	Possible	Consolidation with ground glass opacities	Not performed due to thrombocytopenia	Not done	Negative	Positive	

^*a*^ Microbial cell-free DNA was performed by Karius using plasma (Redwood City, CA).

^*b*^ Panfungal PCR was tested using paraffin block at the University of Washington (Seattle, WA).

### *Nocardia* PCR from BAL

BAL *Nocardia* PCR was tested in 85 of 134 (63%) patients, and 4 of 85 (4.7%) tested positive. Three of these four yielded *Nocardia* growth in culture with isolation of *Nocardia cyriageorgica* (*N* = 1) and *Nocardia Africana/Nova* (*N* = 2). It took 4 days for matrix-assisted laser desorption ionization time-of-flight (MALDI-TOF) to detect *N. cyriageorgica,* and it took 6 days and 11 days for MALDI-TOF to detect *N. Africana/Nova*. The remaining patient with a positive *Nocardia* PCR test had a concurrent biopsy that supported an alternate diagnosis of mucormycosis, negative AFB stain, and no growth in culture.

## DISCUSSION

In this study, we examined the performance of a commercially available PCR assay for the diagnosis of invasive pulmonary disease caused by *Aspergillus*, *Mucorales*, and *Nocardia* using BAL samples from 134 high-risk immunocompromised patients with underlying hematologic malignancy and/or HCT. The BAL *Mucorales* PCR test was positive in three of seven (42.9%) proven pulmonary mucormycosis. Notably, cultures were negative for all seven patients with biopsy-proven pulmonary mucormycosis. All patients who grew *Nocardia* from BAL culture were positive with BAL *Nocardia* PCR. BAL *Aspergillus* PCR was positive in only 9 of 20 (45%) patients with probable or proven IPA. Only one of 20 (5%) was diagnosed with IPA solely by BAL *Aspergillus* PCR test.

In our study, the BAL *Mucorales* PCR test was positive in 3 of 7 (42.9%) proven pulmonary mucormycosis. Notably, cultures were negative for all seven patients with biopsy-proven pulmonary mucormycosis. The BAL *Mucorales* PCR test was also positive in two patients with possible invasive fungal disease where concurrent biopsy was not performed due to refractory thrombocytopenia. We cannot estimate how many cases of *Mucorales* were missed by BAL *Mucorales* PCR among those patients without biopsy. Other studies have also demonstrated the utility of the *Mucorales* PCR using a 3 qPCR combination assay targeting major *Mucorales* genera (*Rhizomucor*, *Mucor*/*Rhizopus*, and *Lichtheimia*) for the diagnosis of mucormycosis and co-infection with *Aspergillus* (21–23).

The previously reported sensitivity and specificity of BAL *Aspergillus* PCR range from 57% to 93% and 92% to 99%, respectively (13). This is comparable with the reported performance of BAL galactomannan with a sensitivity of 52%–86% and a specificity of 93%–100% (13, 24). In our study, the BAL *Aspergillus* PCR test was positive in only 9 of 20 (45%) patients. Only one of 20 (5%) was diagnosed with IPA by BAL *Aspergillus* PCR. One reason for this finding could be the effect of mold-active antifungal prophylaxis on the sensitivity of the *Aspergillus* PCR assay since 70% of patients in the cohort were on antifungal agents at the time of bronchoscopy (25). In a previous study by Smith et al. using the same diagnostic test *Aspergillus*, *Mucorales*, and *Nocardia* PCR (Eurofins-Viracor) in a population of various risk factors, including hematologic malignancies, solid cancer, and solid organ transplant, the BAL *Aspergillus* PCR sensitivity was also low (31.25% sensitivity), with a specificity of 97.17%. This report did not assess *Mucorales* or *Nocardia* PCR test performance (20). One patient was diagnosed with IPA by biopsy (case #3 in Table 2). Although pathology was consistent with *Aspergillus*, non-*Aspergillus* hyalohyphomycoses were indistinguishable from *Aspergillus* by morphology (26). BAL *Aspergillus* PCR might be truly negative rather than not detecting *Aspergillus*. Among nine patients with BAL *Aspergillus* PCR positive, BAL *A. fumigatus* PCR was positive in six patients, and none were positive for BAL *A. terreus* PCR. The ability to identify *A. terreus* is important for antifungal treatment decisions as *A. terreus* is resistant to amphotericin. Additionally, *Aspergillus* PCRs in the United States are currently all laboratory-developed tests (LDTs) with variations in testing methodologies from assay design to nucleic acids extraction and PCR amplification targets and protocols. Thus, the lack of standardization across methods makes comparison of analytical and clinical performance difficult (27, 28).

Finally, all patients who grew *Nocardia* from BAL were positive with BAL *Nocardia* PCR. Our data also show improved early detection of pulmonary nocardiosis consistent with findings from other studies that have reported a sensitivity of 88% and a specificity of 74% of *Nocardia* PCR for the diagnosis of pulmonary nocardiosis (29). One patient with BAL *Nocardia* PCR positive had biopsy-proven pulmonary mucormycosis. *Nocardia* PCR from respiratory specimens could detect airway colonization (29).

Fungal culture grew molds in three patients with probable IPA. Case #4 in Table 2 grew *Scedosporium boydii* and *A. niger* in BAL fungal culture, and case #5 grew *Scedosporium boydii* and *A. ustus* in BAL fungal culture. Both had positive BAL galactomannan 3.5 and 1.79, respectively. Case #10 in Table 2 grew *Purpureocillium lilacinum* in BAL fungal culture, and BAL *Aspergillus* PCR was positive. Other fungal pathogens cause invasive disease in this patient population; hence, targeted PCR assays cannot be solely relied upon. Co-infection with more than one fungus has been reported in 23% of patients with hematologic malignancies (30). Polymicrobial infections had higher mortalities compared with monomicrobial infections (22, 31). More studies are needed to explore syndromic panels to detect multiple pathogens in patients at risk of developing invasive pulmonary infections.

A limitation of our study is that test performance was assessed in a clinical setting with high exposure to mold-active agents. Also, this was a retrospective study and that testing for the diagnosis of invasive pulmonary infection was left to the clinician’s discretion. Our method of selecting procedures with positive results had the potential for selection bias. Although *Mucorales* and *Nocardia* PCR improved early detection, the case numbers were low. A concurrent biopsy was performed in a majority of patients (61%), but 39% of the patients did not undergo tissue biopsy. Finally, although we used established criteria for invasive fungal diseases, there is potential for ascertainment bias for other invasive pulmonary infections.

Overall, our study suggests that targeted molecular PCRs for *Mucorales* and *Nocardia* can be a useful adjunct to routine BAL analysis in difficult-to-diagnose invasive pulmonary infections. In our experience, the role of the *Aspergillus* PCR test was limited in retrospective analysis of high-risk HCT and hematologic malignancy patients with widespread use of mold-active prophylaxis.
